# The IMPROV Project: Protocol for Using a Modified Nominal Group Technique to Prioritise Oral Health Promotion Strategies With the Karen Refugee Community in Victoria

**DOI:** 10.1111/hex.70781

**Published:** 2026-07-22

**Authors:** Sudheer Babu Balla, Jyothi Tadakamadla, Santosh Kumar Tadakamadla

**Affiliations:** ^1^ Dentistry and Oral Health, Department of Rural Clinical Sciences, La Trobe Rural Health School La Trobe University Bendigo Victoria Australia

**Keywords:** community‐based participatory research, health promotion, Nominal Group Technique, oral health, refugees

## Abstract

**Issue Addressed:**

Refugee parents often experience barriers when accessing oral health information and dental services for their children, including language difficulties, unfamiliarity with health systems, and limited culturally appropriate resources. These challenges can contribute to poorer oral health outcomes. Participatory approaches may help identify oral health priorities that better reflect community experiences and service needs. The IMPROV project (Intervention Mapped for Promoting Refugee Oral Healthcare in Victoria) aims to address these gaps by engaging the Karen refugee community and key stakeholders in priority setting for oral health promotion.

**Methods:**

This protocol describes the prioritisation phase of the IMPROV project, which will use a modified Nominal Group Technique (NGT). Separate NGT workshops will be conducted with Karen refugee parents and other stakeholders, including dental service providers, interpreters, and settlement service organisations. Prior to the workshops, key findings from earlier qualitative interviews will be summarised and presented to participants. Participants will then generate ideas, discuss them, and independently rank the most important ideas. Quantitative ranking scores and qualitative discussion data will be analysed to identify the most important priorities.

**Results:**

The primary outcome of the NGT workshops will be a list of the highest‐ranked oral health needs and strategies for improving access to oral health information and dental care for Karen refugee families in Victoria.

**Conclusions:**

This protocol outlines a structured, participatory approach for identifying stakeholder‐informed priorities to guide oral health promotion initiatives.

**Patient and Public Involvement:**

Karen refugee parents were actively involved in shaping this study through earlier qualitative interviews that informed the research focus. They will further contribute to the study through participation in the NGT workshops and co‐design sessions to identify priorities and collaboratively develop the intervention. Their lived experiences will inform both the design and interpretation of the study outputs to ensure cultural relevance and practical applicability.

**Abbreviations**
CALD: culturally and linguistically diverse
E2CD: Evidence‐Informed, Experience‐Based Co‐Design
EBCD: Experience‐Based Co‐Design
GRIPP‐2: Guidance for Reporting Involvement of Patients and the Public
IMPROV: Intervention Mapped for Promoting Refugee Oral Healthcare in Victoria
NGT: Nominal Group Technique
PPI: Patient and Public Involvement
QI: qualitative interviews

## 1 Introduction

Oral diseases remain the most prevalent noncommunicable diseases globally, affecting approximately 3.5 billion people, and represent a significant public health burden [1]. The 2022 Global Oral Health Status Report highlights that dental caries, periodontal disease, and tooth loss disproportionately affect socially disadvantaged groups, reflecting broader structural inequities in access to prevention and care [2]. Contemporary public health scholarship further argues that oral health inequities are shaped by social, political, and commercial determinants, rather than solely by individual behaviours [3].

In Australia, oral health care is delivered through a predominantly private system, with over 85% of services provided by private, for‐profit dental clinics, supplemented by public dental services for eligible populations [4, 5]. Access to timely care is influenced by financial capacity, geographic location, and eligibility for publicly funded services [6]. Out‐of‐pocket costs remain a substantial barrier, and public dental waiting lists can be prolonged [6, 7]. National data demonstrate persistent socio‐economic gradients in untreated dental caries, tooth loss, and potentially preventable hospitalisations due to dental conditions [6]. These patterns indicate that oral health inequities are embedded within broader structural arrangements of the health system.

Refugee and humanitarian entrant populations experience additional vulnerabilities that may compound oral health disadvantage. Many arrive in Australia with disrupted access to preventive oral health services, limited prior exposure to organised health systems, and competing settlement priorities [5, 8]. Research conducted with refugee and migrant communities in Australia has identified language barriers, low familiarity with service navigation pathways, financial constraints, and differing oral health beliefs as key determinants of unmet dental needs [9]. For Karen refugee communities resettled in the state of Victoria, we found that these challenges may intersect with varying levels of English proficiency, differences in oral health practices and beliefs, and limited awareness of available public dental services and government support [10]. Traditional oral health promotion approaches that focus primarily on information provision may therefore be insufficient to address these complexities and service utilisation in this population.

Addressing oral health inequities among refugee communities requires more than providing translated information or conventional health education. Experience‐based co‐design methodologies have been proposed as mechanisms to integrate research evidence with stakeholder and community knowledge to produce contextually appropriate, acceptable, and sustainable interventions [11, 12]. Parents play a central role in shaping children's oral health behaviours, assessing healthcare services, and making decisions regarding dental care utilisation. Following resettlement, refugee parents may face additional challenges when navigating unfamiliar healthcare systems, understanding available oral health services, and accessing culturally appropriate oral health information for their children. As a result, engaging parents in the identification and prioritisation of oral health promotion needs is important for the development of family‐centred and culturally responsive oral health interventions.

Participatory approaches, such as co‐design, also known as co‐production, seek to redress traditional power imbalances in health service planning by actively involving service users and key stakeholders in identifying priorities and shaping solutions [13, 14]. It has been increasingly adopted in healthcare settings [15, 16]. For oral health promotion, particularly among culturally and linguistically diverse (CALD) populations, co‐design offers an opportunity to develop oral health strategies that reflect community experiences and are practical for implementation within local service settings.

While participatory approaches offer valuable opportunities for co‐design, there is a need for structured frameworks to guide their implementation, such as the Evidence‐Informed, Experience‐Based Co‐Design (E2CD) framework. E2CD builds on the principles of Experience‐Based Co‐Design (EBCD), which was originally developed to improve healthcare services by systematically integrating patient and staff experiences into quality improvement processes [11]. E2CD extends this approach by explicitly incorporating research evidence alongside lived experience at every stage of decision‐making, thereby strengthening methodological rigour while preserving participatory integrity [17]. The framework positions lived experience at the centre of the research process and proceeds iteratively through interconnected phases that typically include understanding, refining, prioritising, service evaluation and co‐design, and implementation and evaluation. Rather than operating as a linear sequence, E2CD recognises that knowledge generation is relational and cyclical, with movement between phases informed by reflection, dialogue, and emerging insights [17].

Applying participatory co‐design approaches within refugee health research requires careful methodological adaptation to ensure culturally safe and meaningful engagement. In contexts involving culturally and linguistically diverse communities, structured approaches to stakeholder participation may help minimise power differentials, support equitable contribution, and ensure that lived experiences are meaningfully integrated into decision‐making processes. The present study, therefore, adapts the E2CD framework to a refugee oral health promotion context by incorporating staged qualitative exploration, structured stakeholder prioritisation, and collaborative co‐design activities. In doing so, the study seeks to contribute a culturally responsive methodological approach for engaging refugee communities and service stakeholders in the development of oral health promotion interventions.

**Figure 1:**
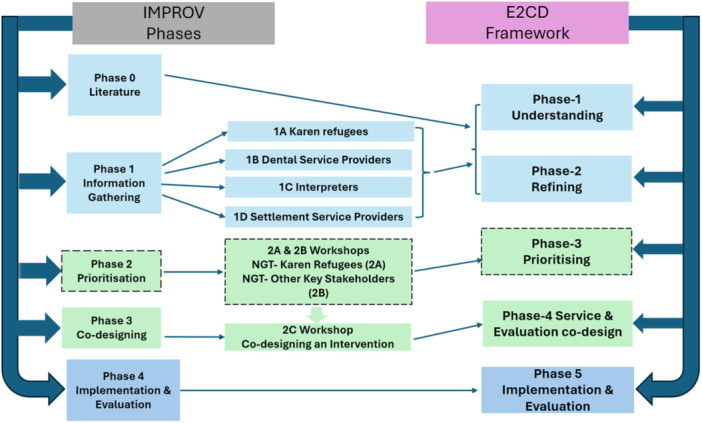
Alignment of the IMPROV research phases with the E2CD framework. Dotted boxes represent the phases specifically addressed within the present protocol paper.

The protocol of the oral health promotion program presented here is the second phase of the Intervention Mapped for Promoting Refugee Oral Healthcare in Victoria (IMPROV) project. The name ‘IMPROV’ reflects the project's goal of improving access to oral health information and dental care for refugee families by identifying community needs and developing practical solutions that reflect the needs and experiences of refugee families. The broader IMPROV project aims to co‐design, develop, and evaluate an oral health promotion program for refugee families. The present protocol specifically focuses on the prioritisation phase of the project, which uses a modified Nominal Group Technique (NGT) to identify and rank stakeholder‐informed priorities that will guide the subsequent co‐design of a culturally appropriate oral health promotion resource. This research adopts the E2CD framework, which combines stakeholder experiences with research evidence to guide the development of culturally appropriate health interventions. Figure 1 illustrates the alignment of the E2CD framework with the research phases.

## 2 Study Aims

This research aims to improve the accessibility, relevance, and cultural responsiveness of oral health information and services for Karen refugee families resettled in Victoria. Specifically, this project seeks to generate an evidence‐informed, community‐endorsed foundation for developing an oral health promotion program that addresses structural, cultural, and system‐navigational barriers experienced by Karen families.

To achieve this, the E2CD framework will be implemented in collaboration with Karen community members and relevant stakeholders, including dental service providers, interpreters, and settlement service providers. The present protocol focuses on the prioritisation phase of the E2CD, which will be conducted using the Nominal Group Technique (NGT) to ensure transparent, equitable, and structured identification of stakeholder‐endorsed priorities. The study brings together community experiences and stakeholder perspectives to identify priorities for improving oral health information and service access among Karen refugee families.

Accordingly, this protocol paper aims to describe the methodological approach used to undertake stakeholder‐informed priority setting within the IMPROV project using a modified NGT. The primary outcome of the study will be the identification and ranking of oral health promotion priorities across stakeholder groups. Specifically, the study will:

1. Conduct separate modified NGT workshops with Karen refugee parents and key stakeholders to identify oral health promotion priorities relevant to refugee families in Victoria.
2. Compare and synthesise stakeholder‐informed priorities across stakeholder groups using quantitative ranking and thematic analysis approaches.

## 3 Methods and Analysis

### 3.1 Study Design

This study adopts a participatory, consensus‐oriented design situated within the broader E2CD framework. Within participatory research, several structured group‐based decision‐making and consensus‐building methods are available, including focus groups and formal consensus techniques such as the Delphi method [18]. Traditional focus groups are designed to facilitate open‐ended discussion and are particularly useful for exploring experiences, beliefs, and perceptions. However, they do not inherently generate ranked or prioritised outputs and may be influenced by dominant voices or existing power imbalances within the group [19, 20].

The Delphi technique, by contrast, uses iterative rounds of questionnaires to obtain convergence of opinion among participants, typically experts [21]. It is well‐suited to geographically dispersed panels and allows for anonymity between rounds [22]. Nevertheless, Delphi studies rely primarily on written feedback, limit direct interaction between stakeholders, and may experience declining response rates across successive rounds. In addition, the emphasis on expert consensus may not align fully with community‐engaged research, where lived experience is central [23, 24].

## 4 Rationale for Using the Nominal Group Technique

Given the objectives of this study, to generate, refine, and transparently prioritise stakeholder‐identified solutions, the Nominal Group Technique (NGT) was selected as the most appropriate method for the prioritisation phase. One of the main reasons for using the NGT is that it is a highly structured face‐to‐face interaction, with substantial work that can be completed in a relatively short time, up to 2 h, suiting participants who are busy engaged in professional duties and researchers who require prompt results [21]. Additionally, NGT could be organised to avoid power imbalances that can influence participation and support equitable participation within stakeholder groups. It could enable separate sessions for community members and health professionals, a more suitable approach when working with CALD populations [25]. The following sections describe the technique in detail and outline its application within this protocol.

## 5 Patient and Public Involvement

Patient and Public Involvement (PPI) is central to this research and reflects a meaningful engagement rather than a tokenistic consultation. Karen community members will be involved not only as participants but as contributors to shaping priorities and informing the design of the proposed oral health resource. This approach aligns with contemporary public health principles that emphasise partnerships with communities to improve the acceptability, equity, and implementation outcomes of interventions [26, 27]. The reporting of PPI activities in this study will follow the Guidance for Reporting Involvement of Patients and the Public (GRIPP‐2) checklist to ensure transparency and completeness [28].

## 6 Participants, Recruitment, and Sampling

This study will be undertaken in collaboration with parents of young children from the Karen refugee community, along with organisations and stakeholders with relevant expertise aligned with the study objectives. These stakeholders will include dental service providers, interpreters, settlement workers, community health service staff, and researchers. Participants will be recruited using purposive sampling with a maximum variation approach to capture a diversity of perspectives across stakeholder groups. Recruitment will occur through established community networks, collaborating organisations, professional contacts, stakeholder referrals, community flyers, and word‐of‐mouth approaches. Karen community participants will be recruited with support from community organisations and trusted community contacts familiar with the local Karen community. Recruitment materials will be provided in Karen language, and interpreter support will be available where required.

Recruitment and participant selection will be guided by the maximum variation sampling matrix presented in Table 1. Recruitment will aim to capture variation in characteristics relevant to oral health access and service navigation, including demographic background, lived experience, professional role, and years of experience where appropriate. The sampling characteristics were selected pragmatically based on observable factors that may influence participants' experiences, service interactions, and perspectives on oral health access and navigation. While these characteristics may not fully determine participant perspectives, they were considered useful for supporting variation in viewpoints across stakeholder groups within the constraints of the study design. Given the relatively small size of the NGT workshops, it may not be feasible to achieve broad representation across every sampling characteristic within each stakeholder group. The sampling matrix will therefore function as a flexible guiding framework rather than a quota‐based sampling structure. Maximum variation sampling will allow the study to identify common patterns across diverse experiences while also highlighting important differences between subgroups [29]. Recruitment and participant selection will occur iteratively throughout the study. Where the expression of interest exceeds workshop capacity, priority will be given to participants who represent characteristics underrepresented in the sampling matrix to support diversity of perspectives within each workshop.

**Table 1:** Maximum variation sampling matrix across stakeholder groups.

Participant group	Priority criteria	Categories
Refugee Parents (Karen parents/carers)	Parent/carer role	Mother/Father/Grandparent or other carer
	Age group	18–29/30–44/45+ years
	Time in Australia	< 2 years/2–5 years/>5 years
	Oral health service utilisation	None in the last 2 years/Occasional/Regular
	Children's age group	0–5 years/6–12 years/Mixed
Dental Service Providers	Professional role	Dentist/Oral Health Therapist/Dental Hygienist
	Practice setting	Public dental/Private practice/Community health/University clinic
	Refugee/CALD caseload exposure	High/Moderate/Minimal
	Years of experience	< 5 years/5–10 years/>10 years
	Interpreter use in practice	Rarely/Sometimes/Frequently
Interpreters/Bilingual workers	Role type	Professional interpreter/Bicultural worker/Community liaison
	Mode of delivery	In‐person/Telephone/Video/Mixed
	Setting commonly engaged	Dental clinics/Hospitals/Community services/Mixed
	Years of experience	< 2 years/2–5 years/>5 years
	Training in health terminology	Formal training/Informal training/None
Settlement Service Providers	Professional role	Case worker/Community engagement worker/Program coordinator
	Organisation type	Government‐funded/NGO/Community organisation
	Client stage	Newly arrived (0–12 months)/Established clients/Mixed
	Program focus	General settlement/Family support/Employment/Health navigation
	Years working with refugees	< 2 years/2–5 years/>5 years

Sample size will be informed by prior EBCD studies and methodological guidance for NGT. Previous EBCD projects conducted within single services have typically included approximately 10 participants for information‐gathering and subsequent co‐design stages [30, 31]. However, as the present study adopts a broader, system‐level perspective involving multiple stakeholder groups, up to 10 participants will be recruited within each stakeholder group to support inclusion of range of perspectives across contexts. With respect to the NGT workshops, there is no universally agreed optimal group size; published reports describe groups ranging from as few as two to as many as fourteen participants [25, 32]. Consistent with methodological recommendations and participatory aims of the present study, each NGT workshop will aim to include approximately 6–10 participants. This group size was considered appropriate to ensure diversity of perspectives while maintaining manageable group dynamics and equitable participation during facilitated discussions. Smaller workshop sizes were also considered particularly important, given the involvement of interpreter‐supported discussions and the need to support culturally safe engagement and meaningful contributions from all participants within the refugee community context [13, 21].

Owing to power differences among stakeholders in the NGT, it has been recommended that, at each meeting, stakeholders be relatively homogeneous in status [25]. To support culturally safe participation and encourage open discussion, the research team will conduct separate homogeneous NGT sessions rather than combining all stakeholders into a single group. Specifically, two separate NGT workshops will be conducted: one involving Karen refugee parents and one involving professional and service stakeholders. This decision is based on the recognition that Karen participants may feel hesitant to speak openly in the presence of professionals or service leaders, despite the structured nature of NGT, which is intended to minimise social dominance. Although separating stakeholder groups is not a mandatory requirement of the NGT methodology, similar approaches have been described in the literature, in which participants were grouped by role or lived experience [32, 33].

However, the research team acknowledges that separating stakeholder groups may reduce, but not eliminate, power imbalances within the workshops. Differences in age, confidence, professional seniority, social position, and lived experience may still influence participation within stakeholder groups. The structured nature of the NGT process, including silent idea generation, round‐robin sharing, private ranking, and facilitator‐led discussion, will therefore be used to minimise the influence of dominant voices and support equitable participation [34]. Facilitators will also actively encourage contributions from all participants and ensure that discussions remain respectful and inclusive throughout the workshops. In the context of refugee health research, this approach could be considered appropriate for supporting meaningful participation and safeguarding community perspectives during the prioritisation process.

## 7 Study Procedure

Ethics approval for this research was obtained from the Institutional Ethics Committee. The study was reviewed and approved by the La Trobe Human Research Ethics Committee [HEC26145]. All participants will receive a detailed explanation of the study's objectives and data protection policies, and informed consent will be obtained from each participant prior to participation.

## 8 Modified Nominal Group Technique Process

In this study, a modified Nominal Group Technique (NGT) will be employed to support priority setting within the co‐design process. NGT is a highly structured face‐to‐face group interaction originally developed to generate ideas and reach consensus through sequential steps [35]. However, in applied health research, the technique is frequently adapted to accommodate the specific aims of the study and the context in which it is used. Variations to classical NGT approach are commonly reported, particularly in relation to how ideas are generated and how consensus is achieved. For example, rather than relying solely on silent idea generation within the workshop, some studies draw on findings from prior research phases, such as literature reviews, exploratory surveys, or qualitative interviews, to inform issues presented for prioritisation [36, 37, 38]. This approach may facilitate broader consultation, establish a shared understanding of the topic, and ensure that prioritisation is grounded in previously identified stakeholder experiences.

In the present study, several modifications were considered necessary to support the participatory and culturally responsive aims of the research. First, synthesised findings from earlier interviews will be presented prior to the idea generation to ensure that prioritisation is informed by the lived experiences and perspectives previously identified across stakeholder groups (Table 2). Second, separate homogeneous stakeholder workshops will be conducted with Karen refugee parents and professional stakeholders to minimise potential power differentials and support culturally safe participation. This approach was considered particularly important given the involvement of culturally and linguistically diverse refugee participants alongside healthcare and service professionals. Third, priorities generated across stakeholder groups will subsequently undergo cross‐group synthesis to identify areas of convergence and divergence while preserving stakeholder‐specific perspectives. These adaptations were therefore implemented to strengthen inclusivity, contextual relevance, and the translation of prioritised outputs into the subsequent co‐design phase.

**Table 2:** Original NGT steps and the Modified Nominal Group Technique (NGT) process used for priority setting in this study.

Original NGT		Modified NGT**	
Steps	Brief description	Steps	Brief description
Step 1: Generating Ides (Silent generation)	Participants are given some time to silently reflect or record their individual ideas in response to a question	Step 1: Presentation of evidence from the earlier research phases	Key themes identified from interviews with all stakeholders will be presented to ensure participants share a common understanding of the barriers and needs.
Step 2: Round Robin	The facilitator concisely records each idea of the participants	Step 2: Silent Idea generation	Participants will individually generate ideas in response to the predefined focus question regarding the type of support or resource.
Step 3: Clarification of ideas	Participants express relative importance of each idea	Step 3: Round‐Robin Sharing and Clarification	Participants will take turns sharing their ideas. The facilitator will record all ideas and guide a short clarification discussion.
Step 4: Voting	Participants privately rate each idea	Step 4: Individual Ranking of Priorities	Participants will privately rank their top 5 priorities. Weighted scores range from 5 (highest priority) to 1 (lowest priority). Aggregate scores are calculated to determine the highest‐ranked priorities.
Step 5: Ranking	Each participant ranks the top five ideas, with the highest receiving 5 and the lowest 1	Step 5: Feedback and group discussion of aggregated rankings	Aggregated ranking results will be presented back to participants within each workshop to facilitate discussion and reflection on the reasons underlying prioritised ideas and differing perspectives.
Step 6: Discussion	Scores for each idea are summed and presented to the group for discussion.	Step 6: Within‐Group Analysis	The research team will compile the ranked outputs from each workshop and summarise the top priorities identified within each stakeholder group.
		Step 7: Cross‐Group Synthesis	Priorities from the two stakeholder groups will be compared to identify areas of convergence and divergence. Votes will not be pooled; rather, findings will be interpreted to maintain the integrity of community perspectives.
		Step 8: Translation into Co‐Design Phase	The highest‐ranked and most feasible priorities will be translated into design briefs that will guide the subsequent co‐design workshop

** Two homogeneous NGT workshops will be conducted to minimise power imbalances: Group 1‐ Karen refugee parents (people with lived experience); Group 2‐ Other key stakeholders (dental service providers, interpreters, settlement service providers).

Prior to the formal NGT workshops, the workshop materials, participant instructions, ranking forms, and persona scenarios will be reviewed with Karen community representatives and Karen language support personnel to assess clarity, cultural appropriateness, and participant comprehension. Feedback from this process will be used to refine workshop procedures and materials, where necessary, before the implementation of the NGT workshops.

Following the clarification discussion, participants will privately select and rank their top five priorities generated during the workshop. Weighted scores will be assigned to each ranked item, with 5 assigned to the highest priority and 1 to the lowest‐ranked item among the selected priorities. Aggregate scores across participants will subsequently be calculated to identify the highest‐ranked priorities within each workshop. The aggregated ranking outcomes will then be presented to participants to facilitate further discussion and reflection on the reasoning underlying prioritised ideas and differing perspectives.

## 9 Data Analysis

**Figure 2:**
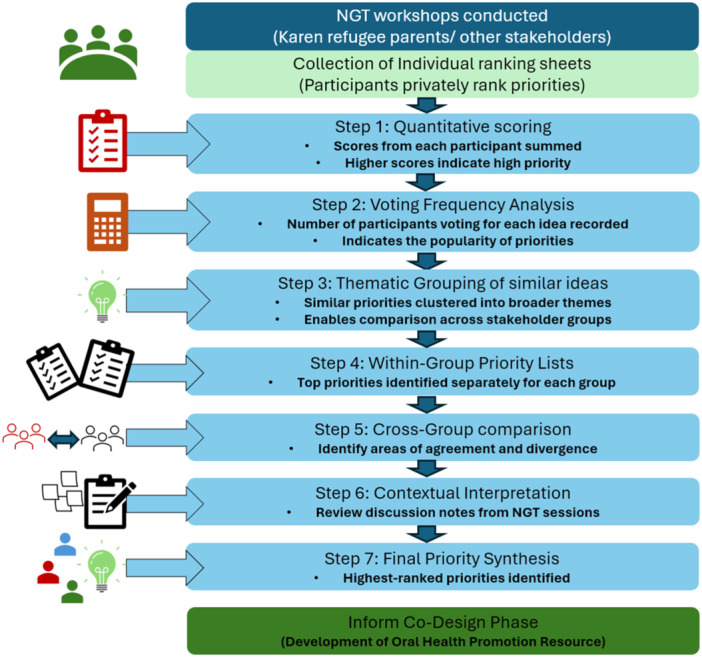
Analytical framework for synthesising priorities generated through the modified Nominal Group Technique.

Data generated from the NGT workshops will be analysed using both quantitative and qualitative approaches (Figure 2). Previous methodological guidance suggests that when multiple nominal groups are conducted, analysis should extend beyond simple ranking within individual groups to allow meaningful comparison across stakeholder groups [25]. Accordingly, priorities identified during the workshops will first be compiled and scored based on participants' rankings. Aggregate weighted ranking scores generated during the NGT workshops will be used to identify and compare highest priority items within and across stakeholder groups. Rather than restricting analysis to only the highest‐ranked items, all ideas generated during the NGT sessions will be considered to understand the relative importance of each issue within the broader set of responses.

To facilitate comparison across groups, similar ideas will be grouped into higher‐order themes through secondary thematic coding. This approach enables researchers to identify common priorities across diverse stakeholder groups while maintaining transparency about how ideas are categorised. Secondary coding and thematic grouping will be conducted independently by two members of the research team to enhance analytical rigour and credibility. The researchers will subsequently compare coding decisions and discuss any discrepancies until consensus is reached regarding the interpretation and categorisation of themes. Where consensus cannot be reached, a third member of the research team will be consulted to help resolve coding differences. As the thematic coding is intended to support the interpretation and synthesis of NGT priorities, rather than establish statistical agreement, formal inter‐coder reliability measures will not be calculated. Qualitative data recorded during the clarification discussions will also be reviewed alongside ranking data to provide a contextual understanding of the reasons underlying participants' rankings and to ensure that thematic interpretation remains grounded in participants' perspectives. Integrating quantitative ranking scores with thematic analysis helps ensure that the final prioritised outputs accurately reflect participant perspectives and provide a robust basis for informing subsequent co‐design activities [25]. The highest‐ranked priorities identified across stakeholder groups will subsequently be synthesised and used to guide the later co‐design phase of the broader IMPROV project [39]. Shared priorities across groups will help identify important areas for resource development, while differences in perspectives will be explored further during future co‐design discussions. Information from the workshop discussions will also help guide the planning and development of culturally appropriate oral health promotion resources and strategies.

## 10 Timeline

**Figure 3:**
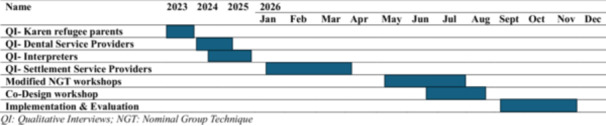
Gantt chart outlining the proposed timeline of IMPROV.

The prioritisation phase of this study will commence with NGT workshops, involving Karen refugee parents and key stakeholders. Two separate NGT sessions will be conducted to generate and rank priority areas. Following the prioritisation phase, the study will proceed to the co‐design phase, during which participants will collaboratively develop a culturally appropriate oral health resource based on the identified priorities. Following the prioritisation phase, the identified priorities will inform a subsequent co‐design workshop involving stakeholder representatives. The later implementation and evaluation phases form part of the broader IMPROV program and are beyond the scope of the present protocol paper. An overview of the study timeline is presented in Figure 3.

## 11 Strengths and Practical Issues

This study has several methodological strengths. First, it adopts a participatory co‐design approach that actively involves both community members and service providers in identifying priorities and developing solutions. Such engagement in research design and decision‐making is widely recognised as a way to enhance the relevance, acceptability, and sustainability of health interventions [31]. Second, the use of the NGT provides a structured and transparent method for generating and prioritising ideas, helping to minimise dominance by individual participants and ensuring that all voices are heard [40]. Third, conducting separate NGT workshops for Karen refugee parents and other stakeholders allows community perspectives to be expressed freely while still incorporating system‐level insights from service providers. Finally, integrating qualitative findings from earlier interviews with structured priority‐setting strengthens the evidence base for the co‐design process and ensures that the resulting intervention is grounded in both lived experience and practical service considerations.

Several practical considerations may influence the implementation of this study. Recruiting participants from refugee communities can be challenging due to competing settlement priorities, work commitments, and potential language barriers, which may affect participation rates and scheduling of workshops. However, the research team has established rapport with the Karen community through earlier phases of this research project and has developed partnerships with local settlement services. These relationships will support participant recruitment by providing culturally appropriate entry points, facilitating communication, and ensuring that recruitment processes are responsive to community needs. Interpreters may also be required to support communication during NGT sessions, which can extend the duration of discussions and require careful facilitation to ensure that participants' views are accurately conveyed. In addition, coordinating separate NGT workshops for different stakeholder groups may require flexibility to accommodate professional commitments and participant availability. Cultural considerations, including building trust and ensuring culturally safe environments, will remain central throughout the research process [41]. A further limitation of this study is that only two homogeneous NGT workshops will be conducted, one with Karen refugee parents and one with professional stakeholders. Although purposive maximum variation sampling will be used to capture a range of experiences and viewpoints, the workshops may not fully capture the diversity of perspectives within the broader Karen refugee community or among stakeholder groups. The findings should therefore be interpreted as representing the views of participants involved in the prioritisation process rather than all members of these populations. Despite these challenges, the use of established community partnerships and structured facilitation methods, such as NGT, is expected to support meaningful participation and reliable priority setting [21].

## 12 Conclusion

In conclusion, this study protocol outlines a structured and culturally responsive approach to identifying and prioritising stakeholder‐informed strategies to improve access to oral health information and care among Karen refugee families in Victoria. Using a modified NGT, the study seeks to ensure that prioritisation is informed by both lived experiences and service‐level perspectives. The findings generated through this study will inform subsequent co‐design activities within the broader IMPROV program and may provide a useful framework for engaging refugee communities in oral health promotion planning and priority‐setting initiatives in other settings.

## Author Contributions

**Sudheer Babu Balla:** conceptualisation, methodology, writing – original draft, writing – review and editing. **Jyothi Tadakamadla:** conceptualisation, supervision, writing – review and editing. **Santosh Kumar Tadakamadla:** conceptualisation, supervision, writing – review and editing.

## Ethics Statement

Ethics approval for this study is currently under review by the La Trobe University Human Research Ethics Committee (HEC26145). All study procedures will adhere to relevant ethical guidelines and regulations. Participants will provide voluntary informed consent prior to participation, and all data will be de‐identified to ensure confidentiality.

## Conflicts of Interest

The authors declare no conflicts of interest.

## Data Availability

Data sharing is not applicable to this article as no data sets were generated or analysed during the current study.
